# Cohort Profile: The Siyakhula Cohort, rural South Africa

**DOI:** 10.1093/ije/dyx148

**Published:** 2017-08-21

**Authors:** T J Rochat, B Houle, A Stein, R M Pearson, M L Newell, R M Bland

**Affiliations:** 1 Africa Health Research Institute, Durban, KwaZulu-Natal, South Africa; 2 Human and Social Development Research Programme, Human Sciences Research Council, Durban, South Africa; 3 MRC Developmental Pathways to Health Research Unit, Department of Paediatrics, School of Clinical Medicine, Faculty of Health Sciences, University of the Witwatersrand, Johannesburg, South Africa; 4 Department of Psychiatry, University of Oxford, Oxford, UK; 5 MRC/Wits Rural Public Health and Health Transitions Research Unit (Agincourt), School of Public Health, Faculty of Health Sciences, University of the Witwatersrand, Johannesburg, South Africa; 6 School of Demography, Australian National University, Canberra, Australia; 7 Institute of Behavioural Science, University of Colorado at Boulder, Boulder, CO, USA; 8 Centre for Academic Mental Health, University of Bristol, Bristol, UK; 9 School of Public Health, University of the Witwatersrand, Johannesburg, South Africa; 10 Global Health Research Institute, Human Development and Health, University of Southampton, UK and; 11 Institute of Health and Wellbeing and Royal Hospital for Children, University of Glasgow, Glasgow, UK

## Summary

Each year millions of children in low- and middle-income countries (LMIC) fail to reach their developmental potential due to factors including poverty, malnutrition, poor stimulation and HIV. Although vertically-acquired HIV can now be prevented, little is known about the impact of HIV exposure in fetal and early life on the development of the many HIV-negative children. The Siyakhula Cohort was established within the Canadian Grand Challenges ‘Saving Brains’ initiative, to support re-enrolment of strategic cohorts in LMIC. This unique cohort in rural South Africa includes 1536 HIV-negative children born to HIV-infected (HIV-exposed) and HIV-uninfected (unexposed) mothers, enrolled from the Africa Health Research Institute (AHRI)–formerly the Africa Centre for Population Health (Africa Centre). The cohort includes data on HIV exposure in fetal and early life, and other early life factors (including breastfeeding) known to impact on later health outcomes. At birth, all children benefited from the early Prevention of Mother-to-Child-Transmission of HIV services in the district, and a subgroup were part of an additional early life breastfeeding intervention, the Vertical Transmission Study (VTS). This cohort pre-dated antiretroviral treatment (ART) roll-out, allowing for examination of outcomes associated with HIV exposure without ART exposure *in utero* and during breastfeeding. Current assessments at ages 7–11 years collected data on growth, health, cognition (including executive function), education and emotional-behavioural outcomes at primary school age. 

## Why was the study set up?

The Africa Centre, one of 11 sites from low-middle income countries (LMIC) to receive funding from Grand Challenges Canada, established the Siyakhula cohort to examine associations between early life factors and later child development, specifically exposure to a breastfeeding intervention^1^^,^^2^ and later child development, allowing for HIV exposure in fetal and early life.

Evidence linking breastfeeding with improved cognition is conflicting, with exclusive breastfeeding (EBF) linked with improved cognition in a meta-analysis^3^ but inconsistent results in a systematic review, depending on study design and methodology.^4^ There is also a dearth of evidence on the effect of HIV on HIV-exposed but HIV-uninfected children. A recent systematic review^5^ examining HIV exposure and child development found data from only 11 studies worldwide (1591 children aged 0–18 years: 650 HIV-exposed; 736 HIV-unexposed; 205 HIV-infected). The review concludes that HIV-exposed children are disadvantaged in terms of child development, in particular emotional-behavioural development, compared with their HIV-negative unexposed peers. However, findings were inconsistent, with most evidence based on small samples with wide heterogeneity in outcome measures. There are few longitudinal studies, almost none with HIV-negative controls or a population norm, and no studies on primary school-aged children.

The Siyakhula cohort was established in 2012 from the Africa Centre [www.africacentre.ac.za] research platform, in a rural, high HIV prevalence setting.^6^ With the scale-up of HIV treatment programmes, parents are surviving to care for their children, and mother-to-child transmission (MTCT) of HIV has been virtually eliminated.^7–10^ Children in the Siyakhula cohort were born in the pre-ART era, between 2001 and 2006, in the Hlabisa sub-district, and are all HIV-negative. Some children had previously participated in the VTS, which supported mothers with exclusive breastfeeding (EBF) for the first 6 months of life, and demonstrated that EBF reduced the risk of MTCT of HIV compared with mixed breastfeeding.^2^ Similar-aged children were also enrolled from the Africa Centre Demographic Surveillance System (DSS). These children had been exposed to the same standard of care, including similar messages regarding HIV and early infant feeding, according to national guidelines at the time,^11^^,^^12^ without the additional VTS breastfeeding support. The Siyakhula cohort is well placed to address the question of whether, in the context of HIV, EBF contributes to improvements in the development and health of children.

## Who is in the cohort?

All children in the cohort are HIV-negative. We excluded HIV-positive children, as they have HIV-specific and unique developmental risks.^5^^,^^13^ Children were eligible for enrolment in the Siyakhula cohort if HIV-negative, 7–11 years of age, born and still residing in the study area (the Hlabisa sub-district), if their mother’s HIV status during pregnancy was known, if mothers received antenatal care for the index child in the study area, and if both mother and child were still alive.

**Figure 1:**
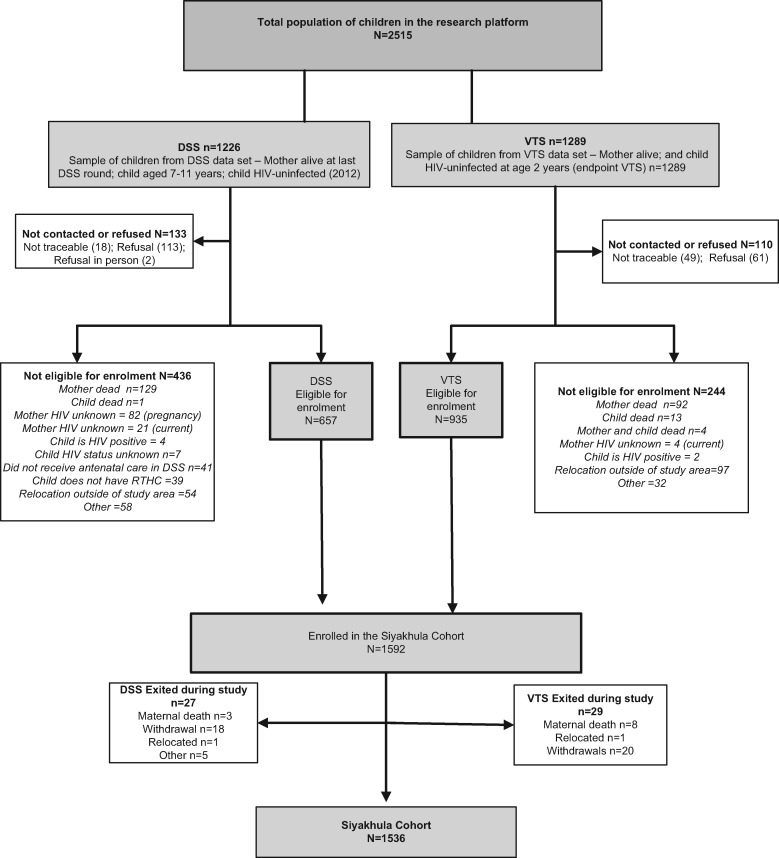
Consort diagram for the Siyakhula Cohort.

**Figure 2:**
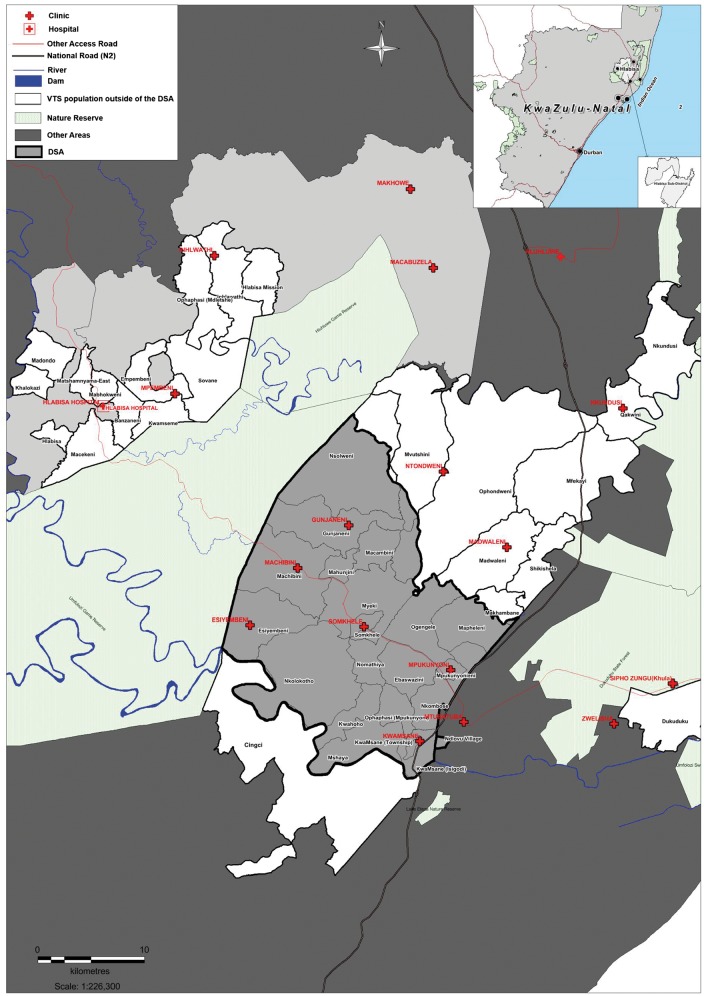

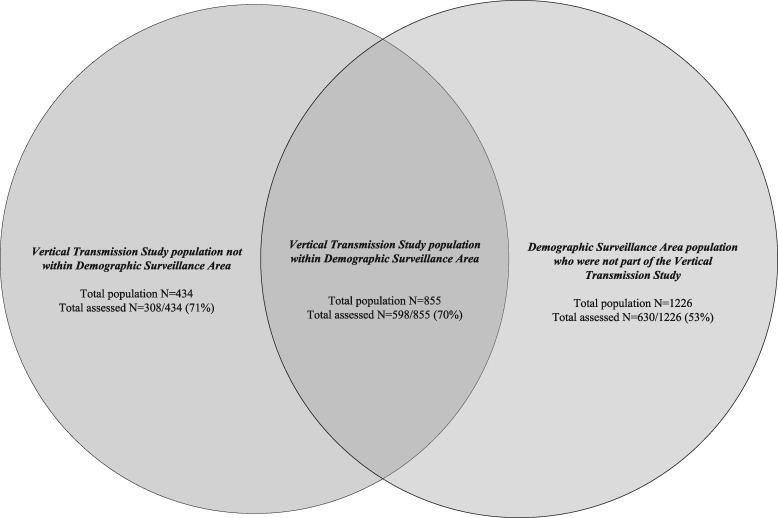
Demographic Surveillance Area within the Hlabisa sub-district, and catchment areas of the Vertical Transmission Study.

The children included in the Siyakhula cohort came from two different sources. First we enrolled HIV-exposed and unexposed children, who met the above eligibility criteria, from the VTS. The children in the VTS had their final study visit when they were 2 years of age. At the end of the VTS in 2006, 1289 children were still alive, were known to have mothers who were alive, and were themselves HIV-negative (see Figure 1). The VTS enrolled children from the Hlabisa sub-district between 2001 and 2005 (see Figure 2).

The second source of children for the Siyakhula cohort came from the Africa Centre Demographic Surveillance Area (DSA), situated in part of the Hlabisa sub-district (see Figure 2).^6^ Since 2000, the Africa Centre has collected data biannually (trianually since 2012) from almost 90 000 people in 11 000 households per round. In 2003, an annual HIV surveillance was added, with HIV status collected from consenting adults.^14^ The children from the DSA had been born between 2001 and 2006, as had those in the VTS, but had not taken part in the VTS (so had not received the EBF intervention). Within the DSA in 2012, 1226 children were documented at their last surveillance visit to be alive, HIV-uninfected, with mothers who were alive (see Figure 1).

It is important to note that all eligible children within the DSA were approached for inclusion in the Siyakhula cohort–some of whom had participated in the VTS and others who had not. However in addition, the Siyakhula cohort included some VTS children who lived in the Hlabisa sub-district but in areas outside the DSA (see Figure 2). Therefore, four groups of HIV-negative children were recruited: HIV-exposed and unexposed from the VTS, and HIV-exposed and unexposed from the DSA. The consort diagram (Figure 1) shows the pool of 2515 potential participants, those who enrolled (*n* = 1592), and those who completed assessments (*n* = 1536). Table 1 shows the characteristics of those who were enrolled compared with those who were not enrolled. Of the 1536 children who completed all assessments, 1059 were HIV-unexposed and 477 HIV-exposed at birth.

**Table 1:** Comparisons of the Siyakhula cohort by enrolled/not enrolled and completed/not completed

	Not enrolled (*N* = 923)		Enrolled (*N* = 1592)		Total (*N* = 2515)		
	*N*	(%)	*N*	(%)	*N*	(%)	*P*-value
Gender							0.009
Female	476	(51.6)	906	(56.9)	1382	(55.0)	
Male	447	(48.4)	686	(43.1)	1133	(45.0)	
Mother’s age at birth							0.035
Less than 20	182	(19.7)	297	(18.7)	479	(19.0)	
20–29	474	(51.4)	755	(47.4)	1229	(48.9)	
30+	267	(28.9)	540	(33.9)	807	(32.1)	
Mother’s HIV status (current)							< 0.001
Negative	158	(30.6)	808	(50.8)	966	(45.8)	
Positive	358	(69.4)	784	(49.2)	1142	(54.2)	
Missing	407		0		407		
Residence at birth							0.949
Rural	575	(62.6)	987	(62.5)	1562	(62.6)	
Urban	343	(37.4)	592	(37.5)	935	(37.4)	
Missing	5		13		18		

	Not completed (*N* = 56)		Completed (*N* = 1536)		Total (*N* = 1592)		
	*N*	(%)	*N*	(%)	*N*	(%)	*P*-value

Gender							0.558
Female	34	(60.7)	872	(56.8)	906	(56.9)	
Male	22	(39.3)	664	(43.2)	686	(43.1)	
Age							0.279
7	1	(3.1)	59	(3.8)	60	(3.8)	
8	4	(12.5)	183	(11.9)	187	(11.9)	
9	17	(53.1)	599	(39.0)	616	(39.3)	
10	6	(18.8)	566	(36.8)	572	(36.5)	
11	4	(12.5)	129	(8.4)	133	(8.5)	
Missing	24		0		24		
Mother’s age at birth							0.021
Less than 20	18	(32.1)	279	(18.2)	297	(18.7)	
20–29	25	(44.6)	730	(47.5)	755	(47.4)	
30+	13	(23.2)	527	(34.3)	540	(33.9)	
Mother’s education							0.405
None	3	(10.0)	92	(6.1)	95	(6.2)	
Primary	8	(26.7)	533	(35.3)	541	(35.1)	
10/matriculation	17	(56.7)	845	(55.9)	862	(55.9)	
Post-matriculation	2	(6.7)	42	(2.8)	44	(2.9)	
Missing	26		24		50		
Birthweight							0.148
<2.5 kg	2	(4.3)	154	(10.9)	156	(10.7)	
2.5 + kg	45	(95.7)	1263	(89.1)	1308	(89.3)	
Missing	9		119		128		
Breastfeeding							0.962
<6 months	16	(34.0)	528	(34.4)	544	(34.4)	
6+ months	31	(66.0)	1008	(65.6)	1039	(65.6)	
Missing	9		0		9		
Birth order							0.261
1–2	22	(68.8)	837	(54.5)	859	(54.8)	
3–4	6	(18.8)	372	(24.2)	378	(24.1)	
5+	4	(12.5)	326	(21.2)	330	(21.1)	
Missing	24		1		25		
Mother’s HIV status							0.443
Negative	20	(41.7)	779	(50.8)	799	(50.5)	
Positive pregnancy	17	(35.4)	477	(31.1)	494	(31.2)	
Positive post-pregnancy	11	(22.9)	278	(18.1)	289	(18.3)	
Missing	8		2		10		
Residence at birth							0.423
Rural	32	(68.1)	955	(62.3)	987	(62.5)	
Urban	15	(31.9)	577	(37.7)	592	(37.5)	
Missing	9		4		13		
Main income provider							0.954
Other	20	(58.8)	907	(59.3)	927	(59.3)	
Mother	14	(41.2)	622	(40.7)	636	(40.7)	
Missing	22		7		29		
Fridge							0.660
No	10	(29.4)	400	(26.1)	410	(26.1)	
Yes	24	(70.6)	1135	(73.9)	1159	(73.9)	
Missing	22		1		23		

‘Enrolled’ refers to children from the original population of children from the VTS and DSS who met the criteria for the Siyakhula cohort and whose parents/guardians provided written informed consent. ‘Not enrolled’ refers to children from the original population of children from the VTS and DSS who were not enrolled for a number of reasons listed in Figure 1, including those who could not be traced, those whose parents/guardians did not provide written informed consent and those who were not eligible for inclusion. ‘Completed’ refers to children who completed all the Siyakhula developmental assessments and other data collected. ‘Not completed’ refers to children who did not complete all the Siyakhula developmental assessments and other data collected.

## How often have they been followed up?

Data for Siyakhula have been collected over three visits between September 2012 and June 2014, when the child was between 7 and 11 years of age. Study consent was obtained in Visit 1, socio-demographic, economic and health data, mothers' mental health and cognitive ability in Visit 2, and children’s cognition and executive function in Visit 3. When the mother was not the primary caregiver, mental health assessments were completed by the child’s primary caregiver during Visit 2. Differences between those lost to follow-up and those who completed assessments are shown in Table 1. Children who are part of the DSS also have longitudinal data available, collected biannually.

## What has been measured?

Table 2 describes the measures used and data collected.

**Table 2:** Description of data collected in Siyakhula

Maternal	Collected from the biological mothers of the Siyakhula enrolled child	Source of data collection
Sociodemographic	Current age	Available from DSS or VTS, and confirmed by maternal report during baseline Siyakhula interview
	Highest educational level achieved	Maternal report in Siyakhula interview
	Current relationship/marital status	Maternal report in Siyakhula interview
	Current income	Maternal report in Siyakhula interview
	Current employment	Maternal report in Siyakhula interview
	Number of surviving biological children	Maternal report in Siyakhula interview
	Current IQ	Tested in Siyakhula using Ravens Standard Progressive Matrices
	Participation with index child in homework, indoor and outdoor activities and meals	Assessed in Siyakhula using the Childhood HOME Inventory, locally adapted 25-item abridged version
Physical health	HIV status	During pregnancy of index child available from DSS or VTS databases. Confirmed by maternal report during Siyakhula interview.Current HIV status by maternal report and confirmed using maternally held clinic health records
	Most recent CD4 count and time of test, where applicable	Maternal report in Siyakhula and confirmed using maternally held clinic health records
	Current ART medication, where applicable	Maternal report in Siyakhula and confirmed using maternally held clinic health records
	Morbidity, including ever had TB or on chronic medication for any other illnesses (e.g. epilepsy or diabetes)	Maternal report in Siyakhula and confirmed using maternally held clinic health records
	Hospitalizations since birth of study child	Maternal report in Siyakhula and confirmed using maternally held clinic health records
	Current height, weight, body fat	Measured in Siyakhula
Mental health^a^	Mental health including depression (PHQ-9) anxiety (GAD-7) alcohol use (AUDIT)	Measured in Siyakhula

Child	Collected from the biological mother (parent-reported)	Source of data collection
Socio-demographic	Gender	Available from DSS or VTS, and confirmed by maternal report during baseline Siyakhula interview
	Birth order	Available from DSS or VTS, and confirmed by maternal report during baseline Siyakhula interview
	Education (crèche and school attendance, absenteeism, repeated grades)	Maternal report in Siyakhula interview
	Biological father	Maternal report in Siyakhula interview
	Biological father vital status	Maternal report in Siyakhula interview
	Biological father financial support	Maternal report in Siyakhula interview
Caregiving	Primary caregiver (education, employment, age if not biological mother)	Maternal report in Siyakhula interview
	Mother, father or primary caregiver involvement in child’s daily routines (including time spent helping with homework, indoor and outdoor activities, meals)	Maternal report in Siyakhula interview, and using Childhood HOME Inventory, locally adapted 25-item abridged version
	Male involvement in child’s life	Maternal report in Siyakhula interview, and using Childhood HOME Inventory, locally adapted 25-item abridged version
	Early child stimulation and discipline	Childhood HOME Inventory, locally adapted 25-item abridged version
	Quality of the parent-child relationship	Parent Stress Index PSI-36, Siyakhula
Physical health	HIV status	All enrolled children were HIV-negative at birth and at the end of breastfeeding. Current HIV status of child by maternal report and using maternally held child clinic cards
	HIV exposure at birth	Available from DSS and VTS databases, and confirmed with mother at Siyakhula interview
	Morbidity, including ever had TB or on chronic medication for any other illnesses (e.g. epilepsy or diabetes)	Maternal report and confirmed using maternally held child clinic health records. Detailed morbidity available on VTS children to age 2 years
	Hospitalizations since birth	Maternal report and confirmed using maternally held child clinic health records
	Immunizations from birth to current age of child	Available from DSS and VTS databases and maternal report, confirmed using maternallyheld child clinic health records
	Current blood pressure, heart rate	Measured in Siyakhula^b^ (at home or child brought to a local clinic or community centre for measurements and developmental assessments)
	Height, weight, body fat, mid upper arm circumference	Measured in Siyakhula^b^
		Birthweights available from VTS and DSS databases
		Detailed anthropometry to age 2 years available from VTS database (including length, weight and mid upper arm circumference)
	Cognition	Kaufmann Assessment Battery for Children, 2nd edn (KABC-II). Measured in Siyakhula.^b^ Children aged 7–11 years
	Executive function	Neuropsychological Assessment, 2nd edn (NEPSY-II). Measured in Siyakhula.^b^ Children aged 7–11 years
	Emotional and Behavioural Problems	Parent Report versions of the Child Behaviour Checklist (CBCL). Assessed in Siyakhula. Children aged 7–11 years
Household	Collected from the biological mother	Source of data collection

Composition	Household structure, composition (resident adults and children), exposure to crime, food insecurity	Maternal report; additional data available in the DSS for those living in the Demographic Surveillance Area
Socioeconomic	Main income provider, access to grant support, household assets index, perception of wealth, exposure to crime (past year)	Maternal report; additional data available in the DSS for those living in the Demographic Surveillance Area


‘Current’ refers to data collected in the Siyakhula cohort data collection. ‘Measured in Siyakhula’ refers to the data collection for this cohort, when children were aged 7–11 years.

^a^Mental health data collected from either biological mother (if she was the child’s primary caregiver) or from primary caregiver of index child if biological mother was not the child primary caregiver.

^b^Measured either at home or, if mother preferred (for example if there was limited space at home), child was brought to a fixed building, for example a clinic or mobile unit.

Child cognition was measured using the Kaufman Assessment Battery for Children 2nd Edition (KABC-II), a validated measure of cognitive development in children aged 3–18 years (Table 3).^15^ The test battery was implemented using the Luria model theoretical approach, well-suited to children in low-income, cross-cultural settings where quality and exposure to school may vary. Eleven subtests were administered, including both verbal and nonverbal tests for all domains. Subtests were scored into four index scales, covering all aspects of cognition and used to calculate a mental processing index (MPI) reflective of general intelligence.

**Table 3:** Description of test battery (including subtests and scales) used in the measurement of child cognition and executive function

Child Cognitive Development: Kaufman Assessment Battery for Children 2nd edn (KABC-II)	
Learning index	Attentional-concentration, coding, storage and sensory integration capacities
Atlantis	Tests associative memory and storing newly learned information (child has to see character and learn a nonsense name and nonsense category)
Atlantis delayed	Tests long-term storage and retrieval, storing and efficiently retrieving previously learned information (child has to remember to nonsense name and category after 30–40 minutes)
Sequential index	Analyse, store and code information received via senses, problem-solving capacities
Number recall	Tests memory span, particularly auditory memory, taking in and holding information, and then using it within a few seconds (tests how many of a string of numbers the child can remember)
Word order	Tests memory span and working memory both visual and auditory. Short-term memory storage, taking in and holding information, and then using it within a few seconds (child has to name and order words in presence of interference stimuli, tests both auditory names and visual objects)
Hand movements	Tests short-term visual memory span and storage, taking in and holding information, and then using it within a few seconds (tests how many in a series of hand movements a child can remember, visual only)
Simultaneous index	Complex simultaneous and successive processing capacities
Rover	Non-verbal problem-solving task using spatial scanning, general sequential reasoning, visualization, (board-like game where child has to visualize the most efficient path, taking into account obstacles and rules, thus maintaining sets and resisting impulses)
Triangles	Spatial relations and visualization, including visual processing, perceiving, storage, manipulating and thinking with visual patterns (child assembles triangle shapes to match a picture of an abstract design)
Block counting	Visual orientation in relation to spatial relationships, measures problem-solving with visual patterns [common to maths achievement (child counts and configures blocks against a picture stimulus where blocks are partially or completely hidden from view)]
Planning index	High-level decision-making, executive processes, planning, self-regulation, complex behaviour
Pattern reasoning	Tests fluid reasoning, ability to solving novel problems by using reasoning abilities such as induction and deduction (child is shown abstract and meaningful stimuli to form logical, linear patterns with one stimulus missing, child selects correct stimuli from a set of 4–6 options)
Story completion	Tests verbal mediation, working memory, planning ability and fluid reasoning, requires determining the sequence to the story, have a general knowledge of the situations which can reflect social and interpersonal capacities (child completes a story by selecting from a set of picture cards (including distractors) and placing missing pictures in their correct place and order)
Knowledge index	Crystallized ability or knowledge within a culture that can be applied effectively linked to learning exposure
Riddles	Tests conceptual inference, requires knowledge and factual information, tests lexical knowledge, general reasoning, language development common to all cultures (child has to point to or name concrete or abstract verbal concepts based on characteristics presented by the examiner)

Child Executive Function: Neuropsychological Assessment 2nd edn (NEPSY-II)	

Attention and executive function	Working memory and attention, inhibition, switching and sorting, problem-solving and self-regulation
Animal sorting	Tests switching, cognitive flexibility, behaviour management, and assesses the ability to formulate basic concepts, to transfer those concepts into action (sort into categories) and to shift (switch) set from one concept to another (child sorts cards into two groups of four cards each, using various self-initiated sorting criteria)
Auditory attention	Tests vigilance, selective and sustained auditory attention in presence of distracting stimuli, is designed to assess selective auditory attention and the ability to sustain it achieving vigilance (child listens to a pre-recorded auditory stimulus of a list of words and touches the appropriate circle in the stimulus book when he or she hears the target word)
Response set	Tests attention, inhibition of previously learned stimuli and autonomic responses, child’s capacity to establish, maintain and change a response set, correctly responding to matching or contrasting stimuli (child listens to a series of words and touches the appropriate circle when he or she hears a target word, thereafter child is asked to inhibit learned response if favoured or a new response)

All these assessments were conducted when children were aged 7–11 years in the Siyakhula cohort. Assessments took place at the child’s home, or if mother preferred (for example if there was limited space at home), child was brought to a fixed building, for example a clinic or mobile unit.

The KABC-II test battery is licensed to Pearson Ltd USA,^15^ and test kits and forms were purchased. All subtests in the Luria Model battery were retained without adaptation, and the administration manual was translated under license from Pearson Ltd, with fees waived.^16^ An expert review team, including the authors of the KABC-II, selected the subtests considered most culturally appropriate and one subtest substitution was made (Atlantis and Atlantis delayed tests replaced Rebus/Rebus delayed tests). One additional subtest Riddles from the knowledge scale was included as a supplementary test of vocabulary and general knowledge.

Three additional subtests to the KABC-II were added to test executive function capacities: working memory, inhibition and switching (Table 3). These subtests were taken from the Neuropsychological Assessment Battery 2nd Edition (NEPSY-II),^17^ also licensed to Pearson Ltd USA.^18^ We used individual subtests in the NEPSY battery (Attention and Executive Function Domain) considered appropriate for focused evaluation of neuropsychological functioning. Test kits and forms were purchased; tests were used in their original format, and auditory stimuli were translated under translation license from Pearson, again with fees waived.^18^

Children’s emotional and behavioural problems were measured using the Parent Report versions of the Child Behaviour Checklist (CBCL) for children aged 6–12 years, which has been validated in over 30 countries including South Africa.^19^^, ^^20^ The CBCL, licensed to the Achenbach System of Empirically Based Assessment (ASEBA), offers a comprehensive approach to assessing adaptive and maladaptive functioning and was used with permission and translation licence.

The CBCL comprises two parts: Part 1 includes a competencies questionnaire, including questions on children’s engagement in academics, sports and hobbies, and the quality of their friendships and sibling relationships. CBCL Part 1 is time-intensive and seldom used in a research context, but mostly provides data for clinical interpretation and treatment. We used an abbreviated version, collecting qualitative data on children’s social and peer competencies which were coded and categorized to be used in quantitative analysis.

The CBCL Part 2 behavioural problems rating scale was implemented in full, including a 120-item rating scale which makes up a composite Total problems score; a high score indicating more problems. The parent rated the child’s behaviour on a three-point scale on a series of symptoms which represent eight psychological syndromes. There are 113 numbered items, but item 56 has 7 subitems on somatic symptoms, making 120 items in total. The items are scored as: 0 = not true (as far as you know); 1 = somewhat or sometimes true; 2 = very true or often true. Some items, if endorsed, include qualitative descriptive answers on the child’s problem behaviour. These descriptions are not used in the scoring system and are of clinical interpretative value only.

In Siyakhula, CBCL scores were normed using multicultural Rating-to-Score norming software (purchased from ASEBA) to produce normed t scores for the Total score, the two subscales and the six Diagnostic and Statistical Manual (DSM) disorders [i.e. Internalizing problems including Affective, Anxiety and Somatic disorders, and Externalizing problems including Attention-Deficit Hyperactivity Disorder (ADHD), Oppositional and Conduct disorders]. Cronbach’s reliability was high (α = 0.94), exceeding the α = 0.75 recommendation for a stand-alone measure.

Research assistants, with 5–7 years of research experience, administered the assessments following 2 weeks’ training. Quality assurance and reliability checks were conducted by two Master’s-level psychology graduates. Rater reliability was assessed against a gold standard assessor for a subsample of 10% of assessments, with reliabilities ≥80% for all assessors throughout the data collection period.

## What has it found?

Analysing the VTS children only, we reported that longer duration of EBF (6 months vs <1 month) was associated with fewer than average conduct disorders, and was weakly associated with improved cognitive development in boys.^21^ In addition, HIV-exposed children performed as well as HIV-unexposed children in the domains examined (cognition and emotional/behavioural development). Maternal intelligence quotient (IQ) was strongly associated with children’s later cognitive development, an interesting finding as maternal IQ is seldom included as a confounding variable in breastfeeding studies, particularly in LMIC.^4^

This is, to our knowledge, the largest cohort of HIV-exposed and unexposed children in Africa who have completed a full battery of cognitive and executive function tests.^5^^,^^22^^,^^23^ The sample size is similar to most normative samples in high-income countries.^15^ We considered it necessary to use a full battery of tests given the absence of normative or reference developmental data in African populations. The approach provides an opportunity for children to perform on at least two subtests in each domain of intelligence, including both verbal and non-verbal tests, substantially reducing the risk that performance is a consequence of test-specific variables, or due to cultural or school exposure.

**Figure 3:**
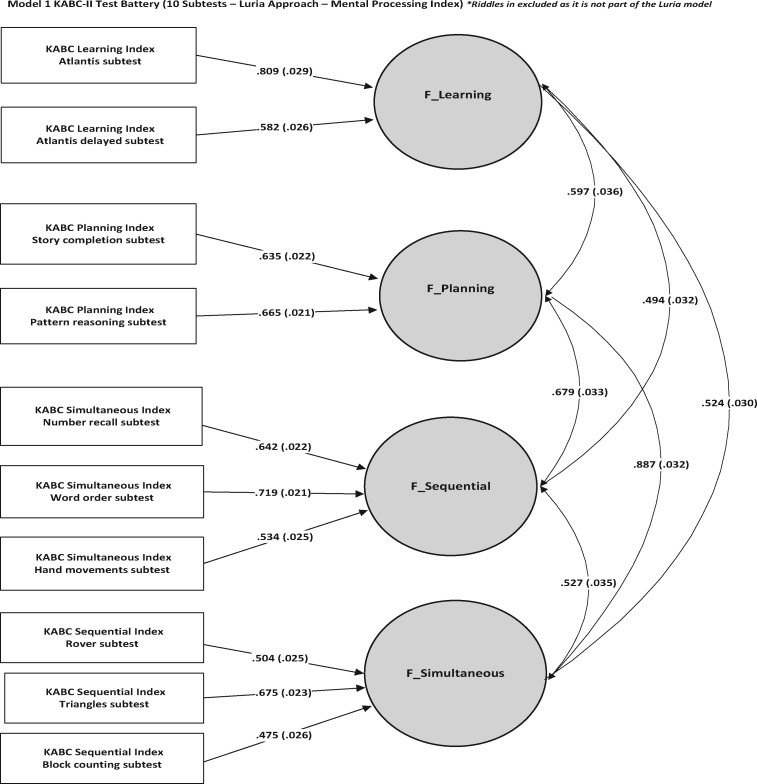

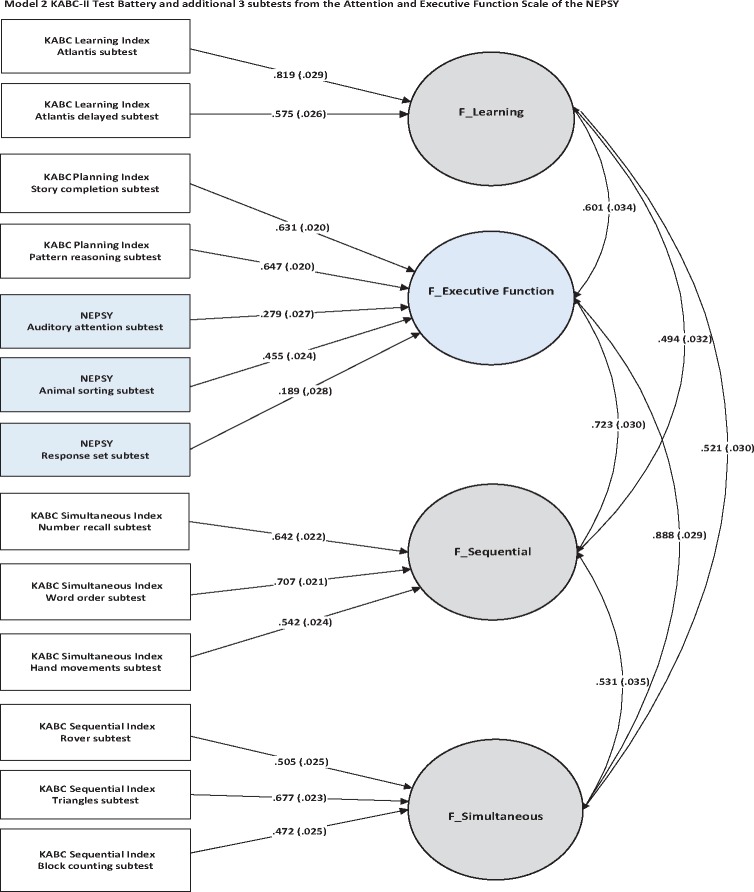
Cognitive model.

We used structured equation modelling (SEM) techniques to test the psychometric validity of the child cognitive measures. This is an important step when using child development batteries in new populations where they have not been tested before. Such techniques also help us to understand the underlying constructs measured by a number of different subtests. The core battery has a pre-determined set of subscales and 10 subtests which are based on well established theories (Luria) of cognitive development. We therefore used confirmatory factor analysis (CFA) to load each specific subtest onto one of four latent factors. Each latent factor represents a domain of cognition. Thus, which latent factor a subtest was loaded onto depended on which of the four domains of cognition the subtest is designed to measure (see Figure 3). SEM is a useful technique to test whether the data in the cohort fit with the expected theoretical model of cognition embedded within the KABC design, i.e. that specific subtests measure one of four key cognitive skills. In addition, SEM techniques separate the construct-related variance from subtest task demands (for example, ability to count or use a pen and paper). This is because the latent variables represent the shared variance across different tasks which measure the same construct in different ways.

We also examined the three NEPSY subtests using CFA and found them to represent individual factors. The NEPSY scales are designed to measure executive function and, in order to improve the KABC factor on planning, we included the NEPSY subscales along with the KABC subscales, combining the NEPSY executive function tests together with the Planning scale of the KABC (Figure 3). The Planning scale reflects a measure of executive functions, hence the factor structure of the test battery reflects a strong battery of both cognition and executive function. The fit of this final model was also acceptable: Comparative Fit Index^24^ (CFI) 0.947, Root-Mean Square Error of Approximations^25^ (RMSEA) 0.047 (0.042, 0.051). Goodness of fit was determined in accordance with Hu *et al.* and was indicated by CFI, Tucker-Lewis fit Index^26^ (TLI) values of over 0.95 and RMSEA of less than 0.06. Multiple indices were used as they provide a more comprehensive evaluation of model fit. The factors were all highly correlated with each other, demonstrating the inter-relatedness of these cognitive capacities and the importance for latent factor modelling to separate out any specific effects.

**Figure 4:**
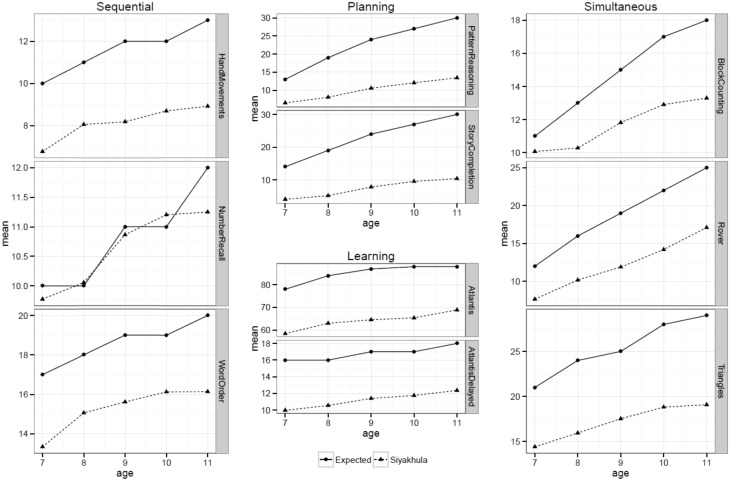
Expected and observed mean scores for the Siyakhula Cohort, by Kaufman Assessment Battery for Children 2nd edn (KABC-II) subtests, and by child age.

In examining children’s performance, Figure 4 shows the mean scores for the KABC subtests by age, comparing expected vs observed scores from the Siyakhula Cohort. Children’s expected scores are derived from the KABC-II normative tables, which indicate the expected score for age at a subtest level.

Overall the developmental scores of the cohort were normally distributed, with the upper tail of the cohort performing in a similar range to the average-to-average children’s scores in high income countries (HIC). However, the vast majority of children in Siyakhula performed substantially lower than their age-equivalent US counterparts. Some of this could be accounted for by variations in school exposure and quality. The differences are nonetheless substantial and widen with age, suggesting that these children would face particular disadvantages within educational settings.

Since children performed poorly across most subtests, one would not expect this poor performance at a scale level to be accounted for by subtest or stimuli effects. One exception is children’s improved performance on the number recall test, in the Sequential scale, which tests a child’s capacity to retain and store information and use it again within a few seconds. The scale has three subtests that use non-verbal hand movements and numerical and verbal stimuli. Children performed well on number recall but consistently poorly on the other two subtests (Figure 4). This likely reflects a higher exposure to number recall tests, common even in poor-quality primary schools. Number recall does not reflect numeracy skills; instead, these are better reflected by performance on the Simultaneous scale. Here children’s performance was again consistently poor across all subtests, although this difference was less marked on the block counting test, perhaps reflecting that rural children were more familiar with the non-verbal stimuli used in this subtest.

The cognitive performance in Siyakhula raises significant concerns for the developmental potential of children in these high-risk populations. Children’s performance on the Learning scale, which reflects how well children learn, store and retrieve new information, critical to educational success, shows that children are disadvantaged from school entry age, and that differences remain large across the age groups. On most scales, with increasing age, children become more disadvantaged, and whereas this reflects the expected cumulative nature of learning or the absence thereof, it also points to the potential advantages of high quality educational interventions in the early years, to ensure that differences are minimized.

## Future plans for the cohort

We are seeking funding to follow the cohort into their adolescent years, to examine development and growth and the effect of EBF on later outcomes. An important area of future research will focus on the development of executive function into adolescence. Executive function is a key area of study across the life course, with poor executive function in childhood predicting early mortality, psychiatric disorders and unhealthy and risky behaviours.^27^^,^^28^ Children’s executive function predicts adult outcomes including employment, low intelligence and low social class origins.^29^^,^^30^ The latter are extremely difficult to modify with interventions, whereas executive function is modifiable to at least 18 years of age.^31^ In Siyakhula, oppositional and conduct disorders emerged as the areas of highest mental health risk and, to lesser extent, child depression. We showed that children’s mental health problems in the areas of conduct disorders were strongly associated with executive functions.^32^ These data provide a key backdrop against which to examine the role of executive functions and mental health as pathways to risk in early adolescence.

## What are the main strengths and weaknesses of the study?

### Strengths

There are almost no large-scale cohort studies in Africa which have used a cross-culturally relevant battery approach to measure children’s cognition in such detail, and very limited data on children’s development in high HIV-prevalent areas. A recent systematic review of the global literature on the effects of HIV exposure on child development found only 11 studies (one from Asia, three from North America, one from Italy and six from Africa) with adequate quality design and measures of standardized cognitive, behavioural and developmental indices.^5^ Across these studies, cognitive performance, behaviour and developmental delay were measured with 15 different standardized scales from 650 HIV-exposed children (including 205 HIV-positive children) and 736 control children. Our cohort of HIV-exposed and unexposed children (1536) includes a larger number of children than all exposed and unexposed children (1386) in the 11 studies published to date. Furthermore, particular critiques of the existing research are directly addressed by our use of a comprehensive cognitive battery, additional subtests in critical areas of cognition such as executive function, examination of emotional and behavioural outcomes and detailed assessment of almost all known confounding factors, including maternal IQ. In addition we have collected data on children’s growth, body fat and blood pressure, all of which predict later health.

Previous studies examining developmental outcomes were unable to adjust for factors known to influence child development, including socioeconomic factors, early infant feeding, HIV exposure and maternal IQ.^4^ A further limitation of previous studies examining developmental outcomes and early breastfeeding, was the inability to quantify the days of EBF accurately, relying on long periods of maternal recall which have been shown to be inaccurate.^33^ We have been able to do this for the children who received the VTS intervention, which applied the most stringent of breastfeeding definitions.^21^

South Africa does have one existing longitudinal panel study, the Birth-To-Twenty cohort of children born in Soweto in the late 1990 s, with data collected from pregnancy to adulthood.^34^ Birth-To-Twenty has contributed enormously to our understanding of human development in South Africa and remains a valuable national resource, but the cohort exists in the societal context within which it took place. Children born in 1990 were not able to benefit from many of the interventions implemented since 1994, such as free health care and the child support grant from birth. In addition, the Birth-To-Twenty cohort is an urban cohort recruited early in the HIV epidemic. The children in the Siyakhula cohort are rural, younger and were born in an HIV-endemic community. Their age and geographical location within an ongoing surveillance platform offer the potential to examine the effects of national interventions such as child support grants, free access to education and water and sanitation on their health. Our cohort thus provides a powerful comparison group, allowing for the investigation of a different time period, geographical location and policy influences on outcomes.

Finally, there is increasing interest in the outcomes of HIV-exposed but HIV-uninfected children, particularly with the introduction of more complex Prevention of Mother to Child Transmission (PMTCT) regimens including maternal ART, during pregnancy and breastfeeding.^35^ The children in Siyakhula were born before HIV treatment was widely available in South Africa, although single-dose nevirapine was administered as part of the PMTCT programme. This cohort includes HIV-exposed children, and provides an important baseline of breastfed children who were not exposed to ART in pregnancy, for future studies on the impact of fetal and early life exposure to ART.

### Weaknesses

Data are not available on women’s mental health during pregnancy or their mental health in the early childhood period, and cannot be inferred from their current measures of depression, anxiety and parenting stress. We have no data on father’s IQ or education–which are likely to influence child outcomes. The relatively long time period between birth and current follow-up may limit our ability to examine moderators or other factors along the pathway between early life and these later outcomes. Finally, although this was a population-based sample with a well defined sampling frame from the DSS, it was a non-random sample and excluded HIV-positive children. More girls than boys, and slightly more children with HIV-negative compared with HIV-positive mothers, were enrolled. Differences between participants who did or did not complete assessments were limited to children born to older mothers being more likely to complete all assessments than children of younger mothers.

## Can I get hold of the data? Where can I find out more?

Information can be obtained freely from the Africa Health Research Institute website for researchers who meet the criteria for access to confidential data [www.africacentre.ac.za]. Those interested should contact Dr Dickman Gareta, Head of the Research Data Management Department, Africa Health Research Institute.

**Profile in a nutshell**

- The Siyakhula cohort, established in 2012, is an observational cohort investigating associations between early life factors (including exclusive breastfeeding and HIV exposure) and later child development.
- The cohort includes 1536 HIV-negative, rural African children aged 7–11 years, including 477 HIV-exposed (born to HIV-positive mothers) and 1059 HIV-unexposed (born to HIV-negative mothers) children.
- The cohort includes a wide range of health and developmental outcomes including cognitive development, executive function, emotional-behavioural development, physical growth and biomarkers, adjusting for a range of current and early life factors including infant feeding, HIV exposure, socioeconomic status, school exposure, maternal IQ and maternal mental health.
- One round of data collection has taken place in the Siyakhula cohort (2012–14), including three data collection visits per child. Further early life data are available on all children, and for children who reside within a large demographic surveillance area, limited additional longitudinal data are available biannually since their birth.
- Information can be freely obtained from the Africa Health Research Institute website for researchers who meet the criteria for access to confidential data [www.africacentre.ac.za], via Dr Dickman Gareta, Head of the Research Data Management Department, Africa Health Research Institute.

## Funding

The Africa Centre for Population Health, now called the Africa Health Research Institute (AHRI), where the research took place is funded by the Wellcome Trust (Grant Numbers: Previous Africa Centre 097410/Z/11/Z; Current AHRI 201433/Z/16/Z). The DSS is co-funded by the South African Department of Science and Technology through the DST/MRC South African Population Research Infrastructure Network (SAPRIN). The original Vertical Transmission Study was funded separately (Wellcome Trust 063009/Z/00/2). The re-enrolment and assessment of the cohort were funded by Grand Challenges Canada, Saving Brains (Grand Challenges 0063‐03). T.R. is supported by the Newton Advanced Fellowship Scheme (AF160108). The support of the DST-NRF Centre of Excellence in Human Development towards data analysis is also acknowledged.
