# Cohort Profile: The Siyakhula Cohort, rural South Africa

**DOI:** 10.1093/ije/dyy009

**Published:** 2018-01-23

**Authors:** T J Rochat, B Houle, A Stein, R M Pearson, M L Newell, R M Bland

First published online: 21 August 2017, *Int J Epidemiol* 2017; 46(6):1755–1756n. DOI: https://doi.org/10.1093/ije/dyx148.

In Figure 3, in both Model 1 and Model 2, the second and third boxes on the left were incorrectly labelled. They should read KABC Planning Index rather than KABC Learning Index.

